# Cognitive-Postural Interference in Multiple Sclerosis

**DOI:** 10.3389/fneur.2019.00913

**Published:** 2019-08-23

**Authors:** Ludivine Chamard Witkowski, Mathieu Mallet, Mathieu Bélanger, Alier Marrero, Grant Handrigan

**Affiliations:** ^1^Vitality Health Network, Dr. Georges-L.-Dumont University Hospital Centre, Moncton, NB, Canada; ^2^Faculté de Médecine et des Sciences de la Santé, Université de Sherbrooke, Sherbrooke, QC, Canada; ^3^Centre de Formation Médicale du Nouveau-Brunswick, Moncton, NB, Canada; ^4^École de Kinésiologie et de Loisir, Université de Moncton, Moncton, NB, Canada

**Keywords:** dual-task, multiple sclerosis, cognition, postural control, balance, cognitive-postural interference

## Abstract

Multiple Sclerosis (MS) is a neurodegenerative disease associated with cognition and balance impairments, which can lead to accidental falls. Postural control requires cognitive resources. This interaction is quantifiable by using the dual-task paradigm. The cognitive-postural interference (CPI) is commonly evaluated through an assessment of the dual-task cost (DTC). The aim of this review was to summarize literature related to process, results and effects of CPI in MS patients. The Prisma statement was used to guide this systematic review. Eligible articles had to include participants with MS for whom CPI was assessed using the DTC. A total of 14 articles meeting inclusion criteria were retained. All studies used the double stance with eyes open for the postural task component. Three types of cognitive tasks were used: Stroop Color–Word Test (SCWT), Word List Generation and Backward Counting. However, cognitive task scores in single or dual task were unavailable in 11 studies, which prevented calculating the DTC for that task. Prioritization instructions were provided in seven studies. Mutual interference was shown in three studies, postural interference in nine and postural facilitation in two. This review highlights the presence of CPI among MS patients. Postural interference usually occurred during dual task while cognitive performance during dual task was rarely reported. Postural task performance does not appear to vary based on EDSS level. We advise authors of future studies to use the SCWT in combination with postural task measure (sway area and postural sway) for DT assessment, with explicit prioritization instructions. Further, the cognitive and postural tasks should be performed in ST and DT and all results should be presented.

## Introduction

Multiple sclerosis (MS) is a chronic inflammatory and neurodegenerative disease of the central nervous system affecting visual, cerebellar, sensory, and motor functions. MS is associated with executive dysfunction and postural impairments and affects quality of life in 85% of patients (1). Imbalance and risk of falling are reported in early MS patients with absence of clinical disability (2). Moreover, 65% of MS patients have some form of cognitive impairment (3).

Postural control is defined as the body's ability to maintain adequate gravity alignment when maintaining an upright posture with voluntary and involuntary movements (4). It is a complex task that requires integration of visual, vestibular, and somatosensory information by the central nervous system (5). Postural control involves specific cortical areas, and attentional and executive dysfunctions are associated with motor disorders (6). There is a physiological relationship between attention, cognition and balance; these functions are, respectively, treated by the frontal lobes, the thalami and the cerebellum, which are linked by a neuronal network (7).

Postural and cognitive disorders were traditionally measured independently in MS patients; however, the simultaneous assessment of postural and cognitive performances demonstrated an interaction suggesting shared attentional resources (8, 9).

The cognitive-postural interference (CPI) is measured by performing a dual task (DT) examination, which involves conducting a postural task along with a cognitive task and comparing performance with that of single-task conditions. The dual-task cost (DTC) is used to quantify the CPI, which represents the percent difference between DT and ST performance (10). Specifically, in cases where higher values indicate better performance, DTC (%) can be calculated as:

(1) $$\begin{array}{r} \text{DTC}\left(\text{\%}\right)=\frac{\left(\text{DTC Score}-\text{ST Score}\right)}{\text{ST Score}}\text{X }100\text{\%} \end{array}$$

and for variables where higher values indicate worse performance as:

(2) $$\begin{array}{r} \text{DTC}\left(\text{\%}\right)=-\frac{\left(\text{DTC Score}-\text{ST Score}\right)}{\text{ST Score}}\text{X }100\text{\%} \end{array}$$

Further, taking consideration of the DTC of both tasks is important when assessing DTC since nine scenarios can occur: (1) No DT interference; (2) Motor Facilitation; (3) Cognitive Facilitation; (4) Mutual Facilitation; (5) Motor-related cognitive interference; (6) Cognitive-related motor interference; (7) Motor-priority trade-off; (8) Cognitive-priority trade-off; and (9) Mutual interference (11).

There is interest in studying the CPI as it provides insight into real-life affectations of postural control challenges for MS patients who experience cognitive and balance disorders. However, the studies that have reported CPI in MS have used methods and inclusion criteria that differ and may present diverging results.

Two systematic reviews on the CPI were published in recent years (9, 12). However, these reviews included studies published before 09/01/2014 (9) or between 01/01/2005 and 31/10/2015 (12). As CPI in persons with MS is a potentially useful clinical outcome, it is important to have the most recent information to aid further research into CPI as a clinical outcome measurement (9, 12).

The objective of this systematic review was to compare and contrast clinical studies that have assessed CPI in MS patients, including radiologically isolated syndrome (RIS) patients and clinically isolated syndrome (CIS) and to summarize evidence emerging from these studies.

## Methods

This review is based on the Preferred Reporting Items for Systematic Reviews and Meta-Analyses (PRISMA) recommendations, and the search strategy—including keywords and choice of databases (13)—was developed in collaboration with an experienced librarian. Searches were conducted in PubMed, ScienceDirect and SPORTdiscus for potentially relevant studies without date restrictions (last updated in October 2018). The keywords applied for this search were {“Multiple Sclerosis”} AND {“Dual Task” OR “Dual Task Cost” OR Cognitive-Motor Interference”} AND {“Balance” OR “Posture”}. Previous reviews were excluded but examined to identify publications that may have been missed by our search strategy. Reference lists of eligible studies were also reviewed to ensure that all potentially eligible studies were retrieved. Eligible studies were peer-reviewed and original studies, published in either English or French, whose MS participants were performing postural (i.e., double stance, eyes open) and cognitive tasks at the same time. All types of quantitative study designs were eligible for this review.

The exclusion criteria were: (1) studies concerning walking; (2) studies concerning effectiveness of rehabilitation process; and (3) studies concerning effectiveness of medications, unless the pre-treatment data were available.

Once duplicates were removed, titles and abstracts were assessed independently by two reviewers (LCW and MM). The full texts of articles considered potentially eligible were then assessed. Any disagreement between the two reviewers led to a discussion and group-based decision about study inclusion. Results extraction was also conducted by two independent reviewers (LCW and MM). Quality assessment was performed with two tools, the National Service Framework Typology of Evidence—Quality Assessment (NSFTE—QA) (14) and the Quality Assessment of Diagnostic Accuracy Studies 2 tool (QUADAS-2) (15). For the QUADAS-2 tool, we applied it in the same manner that was described by Learmonth et al. (12), for the assessment of the judgement of bias for patient selection, index test and flow of the participants. We modified our judgment of bias for the reference standard element to whether or not there were baseline measures of the ST for each of the cognitive and motor tasks for each study. Study type, methodological information, inclusion criteria, number of MS and control participants, Expanded Disability Status Scale (EDSS) score, type of cognitive and postural tasks, and performance scores in single (cognitive and postural) and in dual task were collected. The DTC for the MS participants was calculated for each study. When a large amount of posture and cognitive data were available in an article, the sway area and the Stroop color and word test (SCWT) (16) were used in the DTC calculation, as they were the most frequently used tests, which facilitated data comparison. It is important to note that the DTC values were calculated using the group average performance of the single and dual task (cognitive and postural). The DTC was then represented on a graph to classify CPI patterns. A balance variable was presented on the X-axis, where a positive result was indicative of improved balance (reduced sway area) during the DT and a negative result would show impaired balance (increased sway area) compared to the ST. Similarly, a negative to positive continuum was used to represent the cognitive variable on the Y-axis (10).

## Results

**Figure 1:**
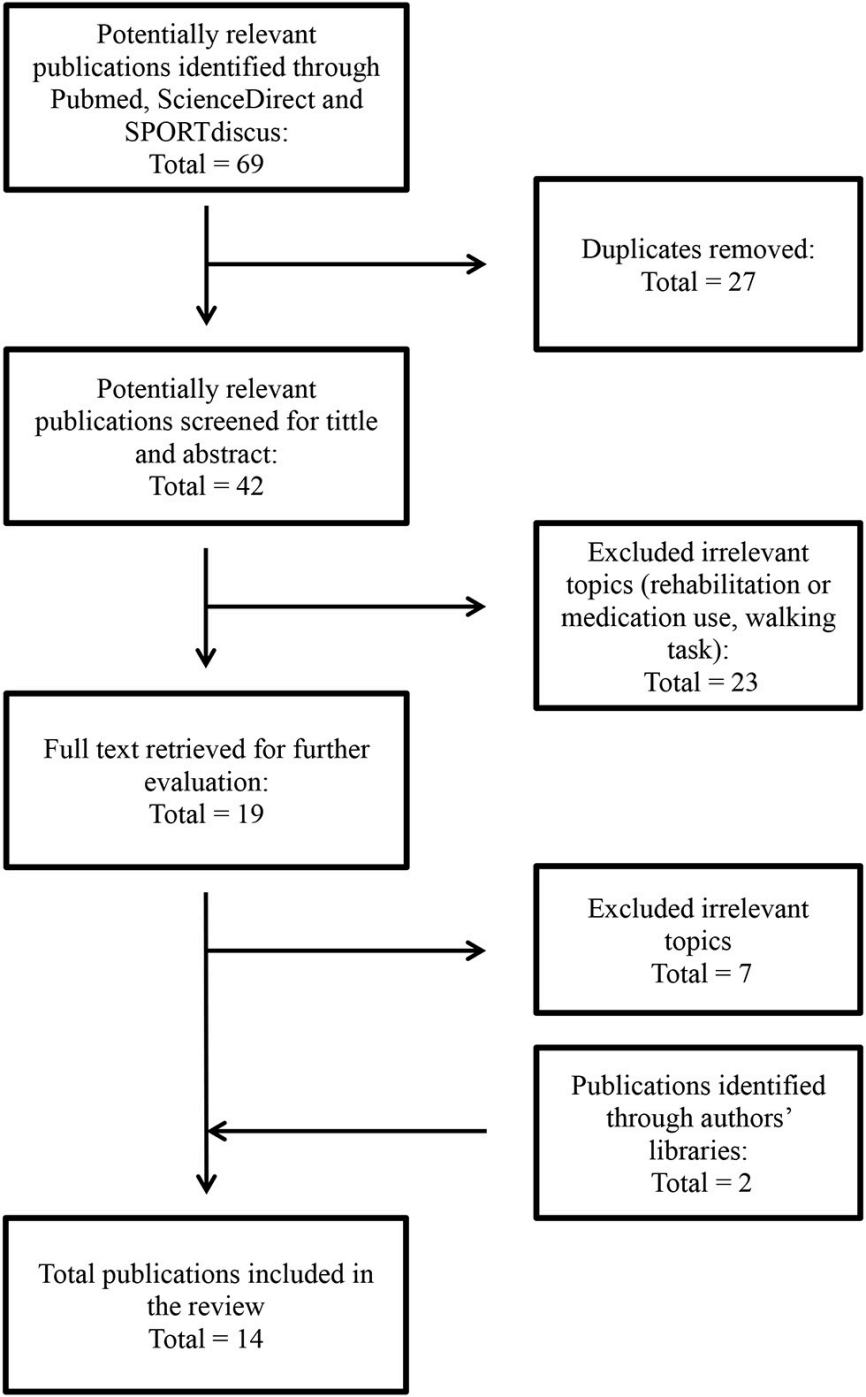
Flowchart of the systematic literature search. The figure was inspired by Moher et al. (13).

A total of 42 distinct references were found. Of these, 23 were excluded after reading the titles and abstracts, and seven more were excluded once they were read in full. Two additional articles were then added to the list following verification of authors' libraries, for a total of 14 articles included in this literature review (Figure 1).

The 14 manuscripts were published between 2010 and 2018 (Table 1). Most of these studies (nine) were cross-sectional and seven had a control group of healthy subjects. One publication had a patient group consisting of subjects with CIS (22) and one with RIS (20), the other publications had subjects with clinically defined diagnostics of MS. The type of MS was not always specified; three articles had patients with relapsing-remitting MS (21, 23, 24) and one with relapsing-remitting and secondary progressive MS patients (26). The inclusion criteria of the MS group were heterogeneous. Most studies had broad inclusion criteria in terms of disability (7, 23–27). Some studies involved patients with an already advanced level of disability, in terms of high EDSS score (4.0–6.5 for the moderate disability group) (17), or risk of falling evaluated with the number of falls during the previous year (21, 28, 29) or moderate to severe spasticity (19), whereas others included patients with low disability represented by low EDSS score (3.0 or less) (18); radiologically (20) or CIS (22). One study (17) compared two distinct groups of MS patients by their degree of disability (Mild vs. Moderate). Some studies excluded patients over 40 years old (28), 45 years old (21, 22) and 55 years old (7, 26, 27). One study (29) involved older patients (50–75 years old). Most studies included patients who had been stable for 1–3 months, could stand upright, and were free of major visual or cognitive impairment (7, 18, 21, 26, 27). Some studies excluded patients with severe depression (7, 18, 26, 27).

**Table 1:** Summary of the characteristics of the studies included in the review.

**ID**	**Study**	**Level of evidence** **/study design/participants/** **inclusion criteria**	**EDSS**	**Cognitive task/** **ST performance/** **DT performance**	**Posture task/** **ST performance/** **DT performance**	**Cognitive** **DTC**	**Postural DTC (double stance with eye open)**	**Prioritization** **instruction/** **DTC calculus**	**Quality**	
									**NFSTE**	**QUADAS-2 (high risk domains only)**
A	Boes et al. (17)	Level II Cross-sectional *N* = 45 adults with MS diagnosis, EDSS 6.5 or less and Relapse-free for 30 days Mild disability group, *n* = 19 (46.4 (13.1), F: 17, M: 2) Moderate disability group, *n* = 26 (58.2 (7.5), F: 24, M: 2)	Median Total 4.0 [2.0–6.5] Mild 3.0 [2.0–3.5] Moderate 6.0 [4.0–6.5]	**Verbal fluency (semantic and phonemic) (# words/30 s):** ST and DT performance not described	MS Total: **Sway area (mm**^**2**^**):** ST: 200.6 DT: 277.3 **Median Velocity-AP (mm):** ST: 6.8 DT: 8.6 **Median velocity-ML (mm):** ST: 8.5 DT: 11.9 **Root mean square displacement-ML (mm):** ST: 5.7 DT: 6.8	Not described	MS Total: **Sway area:** −38.2% **Median velocity-AP:** −26.5% **Median velocity-ML:** −40.0% **Root mean square displacement-ML:** −19.3%	Not described DTC were calculated with the average among the MS group (mild + moderate group)	High	Reference standard
B	Castelli et al. (18)	Level III Cross-sectional *N* = 75 adults (37.8 (9.8), F: 53, M: 22) with MS diagnosis, EDSS 3.0 or less, Stand upright for 180 s, Relapse-free for 3 months, No severe vision impairment, No severe cognitive impairment, No depression	Median 2.0 [0–3.0]	**SCWT (# word's true color/30 s):** ST and DT performance not described	**Postural sway (mm):** ST: 220.0 DT: 332.0	Not described	**Postural sway:** −50.9%	Cognition DTC was calculated with the average among the MS group	High	Reference standard
C	Castelli et al. (19)	Level III Prospective single-group intervention *N* = 22 adults (49.7 (8.3), F: 13, M: 9) with MS diagnosis, Numerical rating Scale ≥ 4, Moderate to severe spasticity, Lack to response to antispastic drugs	Median 5.0 [2.5–6.5]	**SCWT (# word's ink color/30 s)** ST: 22.6 DT: 20.7	**Postural Sway (mm):** ST: 305.0 DT: 456.0	SCWT: −8.4%	**Postural Sway:** −49.5%	Cognition DTC were calculated with the average among the MS group	High	None
D	Dattola et al. (20)	Level II Cross-sectional *N* = 20 RIS group, *n* = 10 adults (33.8 [24–42], F: 7, M: 3) with Okuda's criteria MRI for RIS Control group, *n* = 10 healthy adults (35.0 [29–41], F: 5, M: 5) with no history of known psychiatric or neurological disorders	Non available	**Verbal fluency (semantic and phonemic) (# words/50 s):** ST and DT performance not described	**Sway area (mm**^**2**^**):** RIS-ST: 86.6 Control-ST: 87.2 Semantic Fluency RIS-DT: 105.4 Control-DT: 89.9 Phonemic Fluency RIS-DT: 107.2 Control-DT: 90.6 **Ellipse eccentricity (%):** RIS-ST: 51.6 Control-ST: 52.2 Semantic Fluency RIS-DT: 55.3 Control-DT: 54.1 Phonemic Fluency RIS-DT: 58.3 Control-DT: 54.1 **Sway path length (mm):** RIS-ST: 111.5 Control-ST: 110.8 Semantic Fluency RIS-DT: 145.0 Control-DT: 120.2 Phonemic Fluency RIS-DT: 150.0 Control-DT: 124.1 **Median velocity-AP (mm/s):** RIS-ST: 1.3 Control-ST: 1.3 Semantic Fluency RIS-DT: 2.7 Control-DT: 1.5 Phonemic Fluency RIS-DT: 2.9 Control-DT: 1.5 **Median velocity-ML (mm/s):** RIS-ST: 1.4 Control-ST: 1.4 Semantic Fluency RIS-DT: 2.8 Control-DT: 1.6 Phonemic Fluency RIS-DT: 2.9 Control-DT: 1.6	Not described	**Sway area:** Semantic Fluency RIS: −21.7% Control: −3.1% Phonemic Fluency RIS: −23.8% Control: −3.9% **Ellipse eccentricity:** Semantic Fluency RIS: −7.2% Control: −3.6% Phonemic Fluency RIS: −13.0% Control: −3.6% **Sway path length:** Semantic Fluency RIS: −30.0% Control: −8.5% Phonemic Fluency RIS: −34.5% Control: −12.0% **Median velocity-AP:** Semantic Fluency RIS: −107.7% Control: −15.4% Phonemic Fluency RIS: −123.1% Control: −15.4% **Median velocity-ML:** Semantic Fluency RIS: −100.0% Control: −14.3% Phonemic Fluency RIS: −107.1% Control: −14.3%	Not described DTC were calculated with the average among the two groups	High	Patient selection, Index test, Reference standard
E	Etemadi et al. (21)^*^	Level II Prospective cohort. *N* = 60 adults with RRMS diagnosis, Age 20–45 yo, Stand upright for 60 s, Relapse-free for 1 month, Walk 100 m, No severe vision impairment, No severe cognitive impairment Group ≤ 1 fall, *n* = 34 adults (39.2 (5.1), F: 22, M: 12) Group ≥ 2 falls, *n* = 26 adults (41.7 (6.3), F: 16, M: 10)	Mean Group ≤ 1 fall: 4.0 (0.5) Group ≥ 2 falls: 5.0 (2.0)	**Silent backward counting in multiples of seven (correct response rate):** MS-ST: 36.83 ST and DT performance not described	**Sway area (mm**^**2**^**):** MS-ST: 295.5 DT performance not described	MS: −3.4%	MS: −12.4%	Not described DTC were indicated in the article	High	None
F	Kalron et al. (22)	Level II Observational Case Control *N* = 80 CIS group, *n* = 52 adults (35.2 (1.3), F: 36, M: 16) with CIS diagnosis, Age 20–45 yo, 90 days after onset of symptoms, 1 month free of steroid therapy Control group, *n* = 28 healthy adults (32.8 (1.2), F: 20, M: 8)	Mean 1.7 (0.2) [0–5.0]	**SCWT (# word's true color/30 s):** ST and DT performance not described	**SD of the ML movement:** CIS-ST: 27.0 Control-ST: 13.9 CIS-DT: 37.0 Control-DT: 20.9 **SD of the AP movement:** CIS-ST: 56.0 Control-ST: 41.3 CIS-DT: 57.2 Control-DT: 40.1 **Plane SD (mm):** CIS-ST: 63.8 Control-ST: 44.0 CIS-DT: 69.2 Control-DT: 46.2 **Sway Rate (mm/s):** CIS-ST: 7.9 Control-ST: 4.7 CIS-DT: 11.3 Control-DT: 6.3	Not described	**Standard deviation of the ML movement:** CIS: −37.0% Control: −50.4% **Standard deviation of the AP movement:** CIS: −2.1% Control: +2.9% **Plane standard deviation:** CIS: −8.5% Control: −5.0% **Sway Rate:** CIS: −43.0% Control: −34.0%	Both DTC were calculated with the average among the two groups	High	Patient selection, Index test, Reference standard
G	Negahban et al. (23)	Level II Cross-sectional *N* = 46 MS group, *n* = 23 adults (32.7 (7.9), F: 15, M: 8) with RRMS diagnosis, EDSS 5.0 or less, Stand upright for 30 s, Walk for 100 m, No severe vision impairment, No severe cognitive impairment Control group, *n* = 23 healthy adults (31.4 (7.9), F: 15, M: 8) matched with the patients according to gender, age, height, body mass index, years of education, and MMSE	Mean 2.5 (1.1)	**Silent backward counting in multiples of three (# of subtracting item):** ST and DT performance not described	**Mean total velocity (cm/s):** MS-ST: 1.2 Control-ST: 1.1 MS-DT: 1.2 Control-DT: 0.9 **Sway area (cm**^**2**^**):** MS-ST: 3.4 Control-ST: 2.2 MS-DT: 2.6 Control-DT: 1.0 **SD velocity-AP (cm/s):** MS-ST: 1.4 Control-ST: 1.3 MS-DT: 1.4 Control-DT: 0.9 **SD velocity-ML (cm/s):** MS-ST: 1.7 Control-ST: 1.5 MS-DT: 1.5 Control-DT: 1.3	Not described	**Mean total velocity (cm/s):** MS: 0% Control: +18.2% **Sway area (cm**^**2**^**):** MS: +23.6% Control: +54.6% **SD velocity-AP (cm/s):** MS: 0% Control: +30.8% **SD velocity-ML (cm/s):** MS: +11.8% Control: +13.3%	Both DTC were calculated with the average among the two groups	High	Reference standard
H	Negahban et al. (24)	Level III Prospective single-group intervention *N* = 38 adults (36.0 (8.0), F: 30, M: 8) with RRMS diagnosis, EDSS 2.0 to 5.5, Able to stand without any support, Walk for 100 m, No severe cognitive impairment	Mean 2.8 (1.09)	**Silent backward counting in multiples of seven (# of subtracting item):** ST and DT performance not described	**Mean velocity (cm/s):** ST: 1.7 DT: 1.5 **SD velocity-AP (cm/s):** ST: 0.8 DT: 0.7 **SD velocity-ML (cm/s):** ST: 1.0 DT: 0.9 **SD amplitude-AP (cm):** ST: 0.5 DT: 0.5 **SD amplitude-ML (cm):** ST: 0.6 DT: 0.6 **Area (cm**^**2**^**):** ST: 11.2 DT: 9.9	Not described	**Mean velocity:** +11.8% **SD Velocity-AP:** +12.5% **SD velocity-ML:** +10.0% **SD amplitude-AP:** 0.0% **SD amplitude-ML:** 0.0% **Area:** +11.6%	Cognition DTC were calculated with the average among the MS group	High	Reference standard
I	Porosinska et al. (25)	Level II Cross-sectional *N* = 62 MS group, *n* = 32 adults (33.5 (10.6), F: 22, M: 10) with MS diagnosis, Stand upright for 60 s, No severe pain, nystagmus and vertigo, No severe cognitive impairment Control group, *n* = healthy adults (28.0 (9.9), F: 20, M: 10) matched with the patients according to gender and age	Mean 2.4 (1.1) [1.0–4.5]	**Backward counting from 100 to 0:** ST and DT performance not described	**Sway length-ML (scale not specified):** MS-ST: 3.0 Control-ST: 1.9 MS-DT: 3.5 Control-DT: 1.7 **Sway length-AP (scale not specified):** MS-ST: 3.8 Control-ST: 2.9 MS-DT: 4.0 Control-DT: 3.3 **Sway velocity (scale not specified):** MS-ST: 13.7 Control-ST: 9.1 MS-DT: 17.7 Control-DT: 10.2	Not described	**Sway length-ML:** MS: −16.7% Control: +10.5% **Sway length-AP:** MS: −5.3% Control: −13.8% **Sway velocity:** MS: −29.2% Control: −12.1%	Not described DTC were calculated with the average among the two groups	High	Reference standard
J	Prosperini et al. (26)	Level II Cross-sectional *N* = 138 MS group, *n* = 92 adults (39.2 (10.1), F: 60, M: 32) with RR or SP diagnosis, Age 18–55 yo, Stand upright for 180 s, Relapse-free for 3 months, No severe vision impairment, No severe cognitive impairment, No severe depression Control group, *n* = 46 healthy adults (39.3 (9.8), F: 30, M: 16) matched with the patients according to gender, age and education	Median 2.5 [1.0–6.0]	**SCWT (# word's true color/30 s):** ST and DT performance not described	**COP path (mm):** MS-ST: 298 Control-ST: 198 MS-DT: 405 Control-DT: 231	Not described	**COP path:** MS: −36.0% Control: −16.7%	Cognition DTC were calculated with the average among the two groups	High	Reference standard
K	Prosperini et al. (27)	Level II Cross-sectional *N* = 78 MS group, *n* = 52 adults (48.6 (8.8), F: 30, M: 22) with MS diagnosis, Age 18–55 yo, Stand upright for 180 s, Relapse-free for 3 months, No severe cognitive impairment, No severe depression, No major medication change in the last month Control group, *n* = 26 healthy adults (46.4 (8.1), F: 15, M: 11) matched with the patients according to gender and age	Median 3.0 [1.0–5.5]	**SDMT (# correct symbols/30 s):** MS-ST: 13.8 Control-ST: 18.8 MS-DT: 13.6 Control-DT: 17.7 **Verbal fluency-semantic (# words/30 s):** MS-ST: 18.1 Control-ST: 22.0 MS-DT: 16.7 Control-DT: 21.4 **SCWT (# word's ink color/30 s):** MS-ST: 19.5 Control-ST: 27.9 MS-DT: 18.2 Control-DT: 26.3	**Postural Sway (mm):** MS-ST: 300 Control-ST: 209 **Symbol digit modalities test:** MS-DT: 346 Control-DT: 246 **Verbal fluency-semantic:** MS-DT: 393 Control-DT: 262 **SCWT:** MS-DT: 445 Control-DT: 285	**SDMT:** MS: −1.5 % Control: −5.9% **Verbal fluency-semantic:** MS: −7.7% Control: −2.7 % **SCWT:** MS: −6.7% Control: −5.7%	**Postural sway** **symbol digit modalities test:** MS: −15.3% Control: −17.7% **Verbal fluency-semantic:** MS: −31.0% Control: −25.4% **SCWT:** MS: −48.3% Control: −36.4%	Cognition DTC were calculated with the average among the two groups	High	None
L	Ruggieri et al. (7)	Level II Cross-sectional *N* = 144 MS group, *n* = 96 adults (41.8 (10.6), F: 64, M: 32) with MS diagnosis, EDSS 0–6.5, Age 18–55 yo, Stand upright for 180 s, Relapse-free for 3 months, No severe vision impairment Control group, *n* = 48 healthy adults (40.7 (8.6), F: 32, M: 16) matched with the patients according to gender and age	Median 3.0 [1.0–6.0]	**SCWT (# word's ink color /30 s):** MS-ST: 20.1 Control-ST: 27.8 DT performance not described	**Postural sway (mm):** MS-ST: 308 Control-ST: 198 MS-DT: 427 Control-DT: 245	Not described	**Postural sway** MS: −38.6% Control: −23.7%	Posture DTC were calculated with the average among the two groups	High	Reference standard
M	Wajda et al. (28)^*^	Level III Cross-sectional *N* = 62 adults (60.9 [42–76], F: 46, M: 16) with MS diagnosis, Age over 40 yo Relapse-free for 1 month Fall in the previous year Stand upright for 30 s	Median 6.0 [0–7.0]	**Verbal fluency -semantic and phonemic (# words/30 s):** ST performance not described DT: 12.3	**Sway area (mm**^**2**^**):** ST: 1471.6 DT: 2006.2	Not described	**Sway area:** MS: −93.40%	Not described DTC was indicated in the article	High	Index test, Reference standard
N	Wajda et al. (29)	Level III Prospective single group *N* = 20 adults (61.1 (6.0), F: 18, M: 2) with MS diagnosis, Age 50–75 yo, Relapse-free for 1 month, Fall in the previous year, Walk without assistive device for 6 minutes	Median 5.0 (IQR = 2.5)	**Verbal fluency -semantic and phonemic (# words/30 s):** ST and DT performance not described	**Mean velocity-AP (mm/s):** ST: 15.8 DT: 19.3 **Mean velocity-ML (mm/s):** ST: 9.1 DT: 11.3 **Sway area (mm**^**2**^**):** ST: 1836 DT: 2158 **95% confidence ellipse (mm**^**2**^**):** ST: 938 DT: 877	Not described	**Mean velocity-AP:** −22.2% **Mean velocity-ML:** −24.2% **Sway area:** −17.5% **95% Confidence ellipse:** +6.5%	Not described DTC were calculated with the average among the MS group	High	Reference standard

*Values are represented in mean (± standard deviation) or median [range]. DTC is expressed in percentage. COP, Center of Pressure; DTC, Double Task Cost; MS, Multiple Sclerosis; RIS, Radiologically Isolated Syndrome; CIS, Clinically Isolated Syndrome; RR, Relapsing-Remitting; SP, Secondary Progressive; EDSS, Expanded Disability Status Scale; s, seconds; yo, years old; m, meters; McDonald, McDonald criteria (30) or revised McDonald criteria (31) for MS diagnosis*.

* *The DTC scores for this study were provided directly in the article and they are the values that appear in this table. All of the other values were calculated based on the group means available in the articles. Please see the limitations section for more information*.

The median EDSS score of MS patients included ranged from 2.0 to 6.0, while the average EDSS score ranged from 1.7 to 2.8. The cognitive tasks chosen were tasks that we could qualify as executive function tests. In six studies it was the SCWT, which measures mental flexibility and inhibition capacity (32). Categorical or phonemic word fluencies (e.g., Word List Generation) was used in five studies. These tests measure lexical access as well as mental flexibility (33). Silent Backward Counting with different multiples was used in four studies. This test is more often used to measure the working memory (34). Subjects of one study (27) performed three different cognitive tasks: the SCWT, Word List Generation and the Symbol Digit Test.

For the postural task, participants were evaluated on a force platform where the center of pressure was generated by trials in a double stance with eyes open. Many centers of pressure metrics (e.g., postural sway, sway velocity, and sway area) were generated, but several articles did not have the same center of pressure metrics. Regarding the number of trials, eight studies did only one trial for each postural condition (7, 18–21, 25–27), four studies did two trials (17, 24, 28, 29) and two studies did three trials (22, 23). Concerning the duration of the trials, most of the studies collected the data for a period of 30 s, two studies for 50 s (20, 25) and one for 60 s (21).

**Figure 2:**
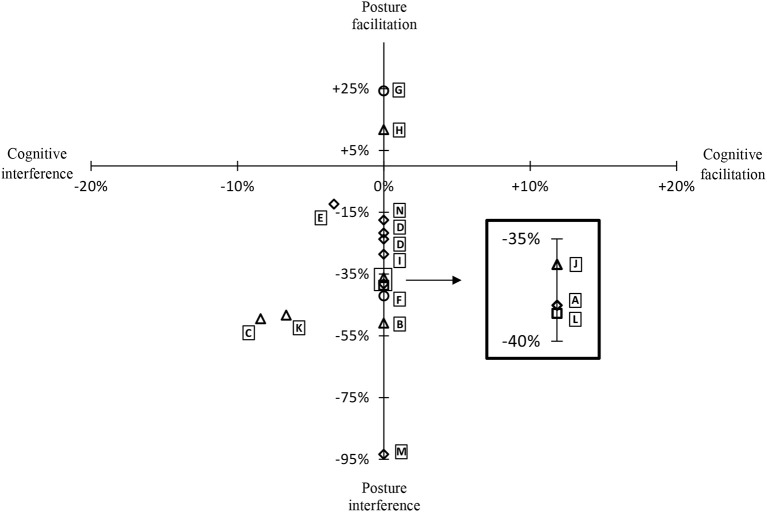
Graphical representation of the DTC (MS subjects) for the included studies of the review. Prioritization instructions: ♢, No instructions, □, Posture, ▵, Cognition, ◦, Both tasks. The DTC from de study of Datolla et al. (20) (D) are represented by two points for the semantic and phonetic fluency tasks.

When it was not directly available in the article, we calculated the cognitive and postural DTC from the available data. The graphical representation of the DTC results from all of the eligible studies is presented in Figure 2. Only three articles had the baseline scores of the cognitive task (during the ST). In these studies, the cognitive/postural DTC showed mutual interference (19, 21, 27). Since the other studies did not present information on baseline score of the cognitive task, their results could not be positioned along the X-axis. Two studies (23, 24) showed a postural facilitation during the DT and the 12 showed a postural interference.

Prioritization instructions were explicitly stated in eight publications, for either the postural task or the cognitive, or both. Five studies (18, 19, 24, 26, 27) had instructed the participants to prioritize the cognitive task and one study asked them to prioritize the postural task (7). Two studies (22, 23) had instructions to prioritize equally both of the tasks during the DT.

Concerning the group differences for the DTC, there were five studies that reported postural interference during the DT condition for both the control groups and the MS groups, with the MS groups showing the largest interference (7, 20, 22, 25, 26). One study reported postural facilitation during the DT condition for both the control group and the MS group, with the control group increasing their performance the most (23). One study used three cognitive tests, and also included the ST cognitive performance scores enabling calculation of the cognitive DTC, and their results differ between the tests: for the SDMT cognitive task DTC for both control and MS groups there was cognitive interference, with the MS group demonstrating less interference, for the verbal fluency and SCWT cognitive tasks DTC for both control and MS groups' demonstrated cognitive interference with the MS group demonstrating greater interference. For this study, the same tendency was found for the postural DTC (27).

## Discussion

In this study, we systematically reviewed methods and results of 14 studies assessing CPI in MS patients. Despite considerable variation in research protocols, most studies documented that DT is associated with a motor related-cognitive interference or motor interference. This signifies that the postural task is affected when the MS patient simultaneously performs a cognitive task. This creates increased falling risks for MS patients in many common situations during activities of daily living, such as standing and talking on the telephone or withdrawing money from an automated banking machine (9).

It would seem that during the DT, the neurological processes associated with postural control and attention share the same neural network in MS patients, which can lead to a deterioration of the postural performance during the DT or vice versa (21), even in a population with no neurological deficit like patients with isolated radiological syndrome (20).

Several theories have been posited to explain the CPI. First, the attentional capacity theory suggests that an individual has a limit to his or her ability to pay attention. During the DT, the capacity is exceeded and one or both tasks' performance will decline (8, 9). Second, the bottleneck theory proposes that due to limited resources, only one task can be performed at a time (35). Third, the self-awareness theory suggests that a person will prioritize one task over the other in a rather conscious way depending on factors such as the environmental demands and task complexity. For example, during the DT, MS patients would alter prioritization of tasks depending on the complexity of the cognitive task and the difficulty of the motor task (36).

This last theory could explain the results of the two studies where there was a postural improvement during the DT (23, 24). It is difficult to know exactly what occurred for the patients of these two studies, as the authors did not present results of cognitive single and double tasks, so we do not know if the cognitive task was improved or worsened by the dual tasking. These publications used a cognitive task considered easier than the ones used in other studies in term of difficulty, however one other study used silent backward counting and found contradictory results (21), that is decreased performance during DT for the MS patients. Furthermore, a recent systematic review and meta-analysis concluded that there are postural control deficits in people with MS when compared to healthy controls (37).

Although the postural task used was similar across studies, the cognitive tasks varied. For example, the SCWT evaluates the executive functions that are most often affected in MS, such as processing speed, long-term memory and attention (3, 38, 39). The SCWT uses a list of names which represent colors printed in the ink of another color (40), and the task consists in reading color names, naming the color of colored dots and naming the intentionally differently displayed color of colors' names. Unlike computation or language tasks, the SCWT is not considerably influenced by level of education. However, mental arithmetic and verbal fluency tasks, as used in other studies, do not require specific materiel and can be used without visual restrictions, which facilitates their use in MS patients with visual impairment. However, it is possible that tasks such as silent backward counting may be too simple for some participants and therefore, the task difficulty varies between participants. It is even possible that such tests allow patients to shift their focus externally and to permit the CNS to perform automatically the postural task more efficiently. A recent study in older adults suggested that an external focus and cognitive task could improve postural control and that could be by the automaticity of sway (41).

Only one study compared various cognitive tasks within their sample population. Prosperini et al. (27) investigated the SDMT, verbal fluency (i.e., semantic) and the SCWT in a sample of 52 adults with MS. They found that there was no main effect on DTC between the three cognitive tasks between the adults with MS and those without MS, with each approximately equally creating a cognitive interference. For the postural task performance, it was found that the SCWT had the largest impact on performance for the adults with MS, when compared with controls; however, their analysis reveals that all of the cognitive tasks created a balance interference in both the adults with MS and those without MS. These results suggest that in order to differentiate those with MS from those without MS for postural performance, the SCWT cognitive task is most effective.

Some studies were interested in MS patients with little or no disability, and those that did investigate found that there was a CPI in the early stage of the disease, such as RIS and CIS patients (20, 22). In this way, the DT paradigm may have the advantage of revealing subtle deficits because of its sensitivity and could even facilitate an early diagnosis of MS. This test challenges the diagnosis of isolated radiological syndrome that is based on the theory that brain lesions are not related to any symptoms (20). That CPI is present in such patients is based on two hypotheses raised by a functional MRI study that indicated lesions could have disconnected two distinct circuits (7), including the anterior and superior corona radiata which connects cerebellum, striatum and pre-frontal areas, and the anterior thalamic peduncles, which connects frontal lobes and anterior/midline nuclear groups of thalami. Accordingly, it indicates that the frontal lobe, which is responsible for executive functions, is related to the cerebellum, which is responsible for balance, but also with the thalamus that contains the sensory pathways used for the pallesthesia. These lesions disconnected the pathways and can cause postural and cognitive impairments during the DT.

The EDSS score seemed to be an important factor to have homogeneous groups, as it was a frequent inclusion criterion. However, one with severely disabled patients did not show more severe interference (29). In addition, another study (17) tested two different groups of MS patients (mild vs. moderate disability) and showed no significant difference in the two groups. Based on these results, we recommend that there should be more research efforts focused on determining appropriate criteria for establishing homogeneity of groups and investigating the differences between EDSS levels and cognitive and motor task performance in persons with MS.

Only three studies (19, 21, 27) presented the results of the cognitive task in single and dual task conditions, thus permitting calculation of the cognitive DTC. These studies showed cognitive and motor interference. Two of these studies (19, 27) prioritized cognition. However, even in these contexts, the two tasks, regardless if they were cognitive or postural, demonstrated performance decrements during dual task performance. Furthermore, it seems that there is less of a difference between the cognitive tests in ST vs. DT than for the postural tests (see Table 1). In other words, the cognitive tasks' performances were negatively influenced, but the postural tasks were even more affected. Therefore, these results demonstrate the importance of standardizing and presenting instructions on task prioritization and also of collecting the task performances in both dual and single task situations.

Concerning group differences, seven studies included a control group thus permitting the comparison between controls and MS participants. For all seven studies the control groups always performed better than the MS participants, thus indicating that the performance of a DT negatively affects the MS groups more than the control groups. However, there was one exception for one specific cognitive task (i.e., SDMT) in the study by Prosperini et al. (27) that showed the opposite effect based on the DTC calculation, with the MS group outperforming the control group for both cognitive and postural tasks. It is important to note that Prosperini et al. (27) did not find the same results as we did likely because of the difference in the manner that the DTC were calculated. For our calculations, we measured the DTC with the group averages because this was the only information available in the published article, and this affects the outcome and interpretation of the DTC results. The limitations section below describes this is more detail.

This systematic review is not without limitations, the primary limitation is in the manner that our DTC scores were obtained. The DTC scores presented in Table 1 were either presented directly in the text of the articles (21, 28) or were calculated based on the information available in each of the articles (7, 17–20, 22–27, 29). When the DTC scores were not available in the articles, the DTCs were calculated by using the group ST and DT means, and it is unclear how much of a difference exists in the DTCs calculated by using the group means compared to using the individual DTC scores to obtain the mean DTC score. For an example of how the two methods can vary, Wajda et al. (28) provided the ST and DT postural scores and provided the DTC mean calculated based on the individual values. When we calculate the DTC using the group means of the ST and DT postural scores, we do not obtain the same value as the DTC score that was calculated with the individual scores. However, this occurred only for this instance and the other articles all used the same method of using the group means to calculate the DTC score. In addition, our objective was to focus solely on postural and cognitive tasks in persons with MS. There are other tasks in activities of daily living that are also of interest to explore the cognitive-motor interferences in persons with MS.

In conclusion, this review highlights the presence of a CPI, whereby impairments in MS patients are associated with postural interference in situations of DT. The level of postural interference was not related to the degree of incapacity as determined by EDSS score. These results suggest that situations when MS patients have to deal with postural and cognitive tasks simultaneously expose them to increased balance impairment, an important precursor of risk of falls. From an assessment perspective, recommendations emanating from this review include that the SCWT appears as the most appropriate cognitive task to use in combination with postural task measure (sway area and postural sway) in the context of DT assessment. Also, DT assessment requires delivery of explicit prioritization instructions to avoid conscious prioritization of one task over another. Further, the cognitive and postural tasks must be performed in ST and DT, and all the results must be presented to provide a clear understanding of CPI affectations.

## Data Availability

All datasets generated for this study are included in the manuscript/supplementary files.

## Author Contributions

MM authored the original draft of this systematic review. LC and GH contributed to the modification of the manuscript for submission. MB and AM provided critical feedback and helped shape the final version of the manuscript. MM and GH contributed equally to the conception of the idea upon which is based this manuscript.

### Conflict of Interest Statement

The authors declare that the research was conducted in the absence of any commercial or financial relationships that could be construed as a potential conflict of interest.
